# Olive Ridley Sea Turtle Hatching Success as a Function of the Microbial Abundance in Nest Sand at Ostional, Costa Rica

**DOI:** 10.1371/journal.pone.0118579

**Published:** 2015-02-25

**Authors:** Vanessa S. Bézy, Roldán A. Valverde, Craig J. Plante

**Affiliations:** 1 Department of Biology, College of Charleston, Charleston, South Carolina, United States of America; 2 Department of Biological Sciences, Southeastern Louisiana University, Hammond, Louisiana, United States of America; University of Regina, CANADA

## Abstract

Several studies have suggested that significant embryo mortality is caused by microbes, while high microbial loads are generated by the decomposition of eggs broken by later nesting turtles. This occurs commonly when nesting density is high, especially during mass nesting events (*arribadas*). However, no previous research has directly quantified microbial abundance and the associated effects on sea turtle hatching success at a nesting beach. The aim of this study was to test the hypothesis that the microbial abundance in olive ridley sea turtle nest sand affects the hatching success at Ostional, Costa Rica. We applied experimental treatments to alter the microbial abundance within the sand into which nests were relocated. We monitored temperature, oxygen, and organic matter content throughout the incubation period and quantified the microbial abundance within the nest sand using a quantitative polymerase chain reaction (qPCR) molecular analysis. The most successful treatment in increasing hatching success was the removal and replacement of nest sand. We found a negative correlation between hatching success and fungal abundance (fungal 18S rRNA gene copies g^-1^ nest sand). Of secondary importance in determining hatching success was the abundance of bacteria (bacterial 16S rRNA gene copies g^-1^ g-1 nest sand). Our data are consistent with the hypothesis that high microbial activity is responsible for the lower hatching success observed at Ostional beach. Furthermore, the underlying mechanism appears to be the deprivation of oxygen and exposure to higher temperatures resulting from microbial decomposition in the nest.

## Introduction

The olive ridley sea turtle (*Lepidochelys olivacea*) is listed on the International Union for the Conservation of Nature (IUCN) Red List as vulnerable and is protected under the U.S. Endangered Species Act (1978) as a threatened species. This species of sea turtle is characterized by a nesting behavioral polymorphism, with some females nesting solitarily and others nesting in mass nesting events called *arribadas* [1]. Hatching success at mass nesting beaches is relatively low (0–32% vs. 74–81% at solitary beaches) and is therefore a concern for the sustainability of this natural phenomenon and the international conservation of the species [2–5]. Current data on the isolated effects of nest density (i.e., competition between developing embryos for respiratory gases and other resources) on hatching success suggest that nest density at *arribada* beaches is not high enough to single-handedly reduce hatching success to the drastically low levels observed [6]. Instead, excessive embryonic mortality may be associated with the particularly high microbial abundance in nest sand resulting from the decomposition of eggs broken by overlapped nesting [2,6,7].

The hatching success of oviparous reptiles is dependent on a complex interaction between the biotic and abiotic characteristics of the nest environment to create a suitable range of conditions for embryonic development. For example, the physical characteristics of the nest substrate (e.g., sand grain size, organic matter content) play an important role in establishing the appropriate diffusion of elements (O_2_, CO_2_, H_2_O, and heat) into and out of the nest cavity, consequently affecting the viability and developmental rate of egg clutches [8,9]. On the other hand, biotic factors such as clutch size and microbial activity have the potential to indirectly affect hatching success by altering nest temperature and oxygen content [10,11].

*Arribada* beaches present a unique nest environment due to the high nest densities and high rates of nest destruction associated with the mass nesting behavior [12]. Because nest density and destruction affect hatching success, these beaches present a temporal and spatial gradient in hatching success that correlates with the spatial distribution and timing of mass nesting events [3]. A study comparing *in situ* nests and hatchery clutches incubated in clean (tidal washed and sieved) sand at an *arribada* beach suggested that high embryo mortality in natural nests was due to higher incubation temperatures and lower oxygen content [11]. Natural tidal washing and the high erosion rates characteristic of mass nesting beaches ensure the replacement of sand and removal of organic matter, which is also believed to be associated with increased hatching success [2,11,13,14]. In fact, a recent study found that while higher bacterial diversity was typically observed in high nest density areas and in association with lower hatching success, the low beach zone (at the same nest density) where frequent tidal exposure occurs did not conform to these trends [13].

While the negative effect of microbial abundance on hatching success has long been presumed [2,6,7], no previous research has ever directly quantified microbial abundance and the associated indirect effects on hatching success at a nesting beach. The particularly high microbial load at *arribada* beaches and its presumed spatial and temporal variability provides a unique opportunity to investigate sea turtle-microbial interactions during embryonic development [2,3,14]. Ostional, Costa Rica is one of the most important olive ridley nesting sites in the world, with mass nesting events estimated at up to approximately 500,000 nesting females over a period of up to seven days [3]. This population of olive ridleys supports a legalized community-based egg harvest program aimed at reducing the number of nests destroyed by subsequent nesting turtles during *arribadas* [2]. There is currently no evidence to suggest that the harvest is having a negative impact on the population [3]. Instead, the apparent decrease in *arribada* nesting population abundance has been attributed to the low hatching success (e.g., as low as 8% in August 1984) at this beach [3,15].

Several studies have examined the presence of microorganisms (such as bacteria and fungi) in association with sea turtle nests. In particular, bacteria and fungi have been cultured and isolated from nest sand and failed eggs as well as from the cloacal fluid of nesting females [14,16,17]. The infection of sea turtle eggs by microbes is commonly thought to be opportunistic and the few relevant laboratory studies to date have found no significant effect of the presence of bacteria or fungi on the hatchling production of olive ridley sea turtle eggs [14,16,18]. However, other studies suggested a negative correlation between bacterial diversity and hatching success [13,17]. Additionally, recent studies on the *Fusarium solani* species complex have identified several fungal pathogens of sea turtle eggs [19,20]. While many studies have identified a diversity of both bacteria and fungi in association with failed sea turtle eggs [16–18], studies examining potential links between embryo mortality to the presence or abundance of microbes are still lacking due to the limitations in obtaining permits and conducting research on protected species as well as the high occurrence of total nest failure in such studies [12,18].

Accordingly, in this study we tested the hypothesis that high microbial abundance was responsible for the low hatching success observed at *arribada* beaches. To test this hypothesis, we treated nest sand to reduce the microbial load. We were specifically interested in exploring treatments that were feasible and applicable to conservation management practices. In order to isolate the relationship between microbial decomposition and hatching success, we monitored nest conditions and hatching success in nests relocated into experimental treatment plots and quantified the microbial abundance in nest sand. Our study supports the hypothesis that high microbial abundance adversely impacts hatching success by altering the nest environment and identifies treatments that are effective at decreasing microbial abundance and increasing hatching success.

## Methodology

### Study Site

The Ostional National Wildlife Refuge (ONWR) is located on the Pacific coast of the Nicoya Peninsula in Costa Rica (9.996471°N; 85.697800°W). Within ONWR, the Nosara and Ostional beaches make up approximately 7 km of beach with variable width. This study was conducted at Ostional beach during the rainy season (May through November) of 2013, when *arribadas* were more abundant. The beach structure is also highly dynamic during the rainy season as nearby estuaries often overflow and cause substantial erosion. The village of Ostional is located adjacent to the main nesting beach, where *arribadas* tend to concentrate [3]. During the 2013 nesting season, however, *arribadas* shifted towards the opposite end of the beach.

### Experimental Treatments

Experimental treatments were applied to plots (50 × 50 cm in surface area and 60 cm in depth) within a high density nesting area, where there was presumably a high microbial load in the sand. Each treatment was replicated ten times. Five additional replicates for each treatment served as “no-nest controls” in which the treatment was applied to the sand but no nest was placed inside the plot. These were used to measure basal oxygen and temperature in the sand exclusive of nests. Control treatments were targeted at isolating the effect of each step in the treatment process on both microbial abundance and hatching success. Treatment plots were positioned using a Latin square block design and separated by a 50 cm buffer zone on all sides.

Removal treatments were applied by removing the sand and letting it soak for 24 hours before rinsing it with freshwater and replacing it. These treatments were specifically designed to alter microbial abundance based on previous studies on the effect of turnover and drying/rewetting on microbial abundance and activity [21–23]. The antimicrobial removal treatment consisted of a 5% dilution of household bleach (6% sodium hypochlorite) in freshwater. Control treatments consisted of a freshwater soak and the removal of sand only. The sand was rinsed by adding freshwater, stirring, and pouring off the flow-through rinse water. Relative chlorine levels of flow-through rinse water were measured to ensure that the sand was properly rinsed until reaching control (freshwater) levels of 0 ppm total chlorine. The sand was then replaced and compacted back into the respective treatment plots.

Topical treatments were applied to the sand by pouring buckets of water (approx. 120 L total) over the surface of the sand, which was enclosed by a wooden frame (50 × 50 × 15 cm) that was partially buried below the surface to ensure that each treatment remained within its respective plot as it was applied. These treatments were specifically designed to minimize labor costs and maximize feasibility for use in conservation management practices. The antimicrobial topical treatment consisted of seawater (25–35 ppt) collected directly from the ocean in front of the treatment area at the nesting beach. Seawater was used here because of concerns regarding the environmental impact of the direct application of bleach to the sand and because its use was supported by previous studies on its effectiveness at altering the microbial community and increasing hatching success [13,24,25]. Control treatments consisted of a topical application of freshwater and no manipulation at all. All freshwater was obtained from an adjacent seasonal estuary (0 ppt during this study).

The removal and topical treatment areas (each being approximately 7 × 8 m) were located directly adjacent to each other in a hatchery above the high tide line. The hatchery was enclosed by fencing and surrounded by large logs to prevent turtles from breaking through during *arribada* events. Nests that were found within this area prior to the study were relocated outside to ensure a controlled nest density throughout. However, organic matter from destroyed nests present in the sand prior to the study was left in place. The entire study area was covered with a black mesh roof (for shade) after day 5 of incubation to protect nests from the onset of the dry season (December-April), which may have threatened successful embryonic development with temperatures above the lethal limit in the latter part of the incubation of our study nests [26].

All study nests were relocated within the first two consecutive nights of the *arribada* that occurred during the last quarter moon of October 2013. This ensured that all nests incubated simultaneously during the study and helped standardize any uncontrollable variables that could affect hatching success, such as ambient temperature and rainfall. Clutches of eggs were collected directly into sterile plastic bags as they dropped from the cloaca of a nesting *arribada* turtle. Eggs were then pooled before haphazardly relocating 100 eggs each into a replicated nest chamber within a 0.25 m^2^ treatment plot until all plots were filled. All nest chambers were constructed to roughly the same dimensions. Cross contamination of the sand was avoided by using a plastic tarp with a 50 × 50 cm hole cut from the center in order to keep the sand that was removed to create the nest chamber separate from the surrounding buffer zone. A datalogger and oxygen tubing were placed within the nest chamber after 50 eggs had been placed. A wire mesh enclosure was placed over each nest on the 40^th^ day of incubation and until hatchlings emerged to prevent predation and to constrain hatchlings within each nest plot as they hatched. From this point on, nests were monitored at least three times daily (sunrise, sunset, and midnight) for signs of hatching in order to count and release hatchings as soon as possible.

### Ethics Statement

The experimental procedures and use of olive ridley sea turtles for this study were approved by the Institutional Animal Care and Use Committee of the College of Charleston (IACUC-2012–021). Field sampling and molecular research permits were granted by MINAET (Resolución No ACT-OR-DR-075–13) and CONAGEBio (R-022–2013- OT-CONAGEBIO) of Costa Rica and authorized both the experimental manipulation of nesting areas and the relocation of study nests.

### Nest pO_2_ and Temperature

The partial pressure of oxygen (pO_2_) in all study nests was monitored by placing an air stone fitted with the tip of 60-cm nylon tubing into the center of the egg-clutch that ran from inside of the nest chamber to the top layer of sand where a shut-off valve impeded any additional gas exchange [6,11,27]. The pO_2_ within the nest was measured using a flow-through oxygen sensor (S108 Oxygen Analyzer, Qubit Systems) that was calibrated prior to the field season using nitrogen and prior to each set of samples using atmospheric air. Dead air space (approximately 10 ml) was drawn up from within the tubing and expelled prior to sampling to ensure the air sample was from within the nest cavity. Air samples (approximately 60 ml) were drawn using an airtight syringe and analyzed within 1 h of collection. Samples were slowly injected through an air pump, flow meter, desiccant column, and through the O_2_ sensor at a flow rate of approximately 50 ml min^-1^. Air samples were analyzed every 5 days for the first 30 days of incubation and every 4 days through the end of the incubation period. Gas percentages were converted to partial pressures using ambient barometric pressure.

Nest temperature was monitored using HOBO pendant temperature dataloggers (Onset Computer Corporation) placed in the center of each egg-clutch and programmed to record temperature at 1 h intervals starting at midnight on the night of oviposition through hatchling emergence. Mean daily nest temperatures were used to compare nest temperatures across treatments.

### Nest Excavations

Ten hatchlings were randomly chosen from each nest and weighed before release (Pesola, Lightline Spring Scale, 0–100 g ± 0.03 g). If fewer than ten hatchlings were observed emerging from the nest, all hatchlings were weighed. Sterile gloves were worn for all excavations and changed between contact with different nests. Nests were excavated the day after hatchling emergence to quantify hatching success by recording the total number of hatchlings that hatched out of their eggshell relative to the total number of eggs originally deposited in the nest [28].

### Sand Collection and Analysis

For the organic matter analysis, a sample of sand was collected from the replicated nest chamber just prior to relocating the nest (hereafter referred to as the initial sampling time point or nest relocation). For all other sediment analyses, a sample of sand was collected directly into sterile collection tubes from the center of the nest chamber during the excavation of the nest (hereafter referred to as the final sampling time point or nest excavation). Samples were placed on ice immediately after collection and either frozen (-20°C) or preserved in formalin (2% formaldehyde) until analysis. The organic matter analysis consisted of a loss-on-ignition method, with the organic matter content being the loss of mass after dry combustion. Water content was also calculated as the loss of mass after drying. The sample was transferred to a porcelain container and desiccated in a drying oven (12+ h at 70°C) before combustion (8 h at 500°C). Combusted samples were pooled by treatment before they were fractionated with a set of sieves (0.063, 0.125, 0.250, 0.500, 0.710, 1.000, 1.400, and 2.000 mm) to determine the particle-size distribution by mass. Mean grain size (ϕ), sorting (σ_ϕ_), and skewness (*Sk*_ϕ_) were calculated using the logarithmic mathematical ‘method of moments’ in GRADISTAT [29,30]. Samples preserved in formalin were used for microscopy counts as a secondary method of quantifying bacterial abundance. These samples were centrifuged for 10 min at 16,000 g in a microcentrifuge before carefully removing excess formalin. The sand was then diluted (approximately 1:2) with sterile water and sonicated on ice for 20 s at 30 W (Sonifier S-250A, Branson). The resulting supernatant fluid was stained for approximately 5 minutes with a 1:10 dilution of 1X SybrGold and sterile water. Microscopy counts were performed at 1000X magnification on an epifluorescence microscope (Optiphot-2, Nikon) by counting 10 fields per slide. The number of cells g^-1^ of sand was then calculated based on the original mass of sand, volume of diluent and supernatant, and the average number of cells field^-1^ using the number of fields per slide at 1000X (4.79 × 10^4^) and a correction factor for the addition of formalin (x1.16).

### Microbial Abundance

We used a quantitative real-time polymerase chain reaction (qPCR) molecular analysis to determine the abundance of bacteria based on the number of 16S rRNA gene copies g^-1^ nest sand and the abundance of fungi based on the number of 18S rRNA gene copies g^-1^ nest sand. While the resulting quantification of gene copies from a qPCR molecular analysis cannot be directly transformed into the number of cells or biomass (given that copy number can vary greatly between species), this can be used as a proxy for overall abundance in microbial communities [31–33].

### DNA Extraction

Each sample of sand was thawed and homogenized by vortexing before collecting a subsample for DNA extraction. DNA was extracted from a 1 g subsample for each nest using a PowerSoil DNA Isolation Kit (Mo Bio Laboratories, Inc.) with a few modifications to the protocol to increase DNA yields. Samples were subjected to 5 minutes at approx. 2,000 oscillations per min in a bead beater (Mini Beadbeater-8, Biospec Products), followed by three freeze-thaw cycles (−20°C and 70°C for 30 min each). Additionally, only 50 μl of the C6 elution buffer was used in the final step, followed by centrifugation and collection of DNA in a sterile tube. For each set of extractions, a negative control DNA extraction was carried out in which no sand was added to ensure no signal originated from the extraction process alone. DNA samples were diluted to reduce inhibition and optimize efficiency and Ct values to within the range of the standard curve.

### qPCR Analysis

Absolute qPCR was run using an iCycler iQ Real-Time PCR Detection System (Bio-Rad Laboratories, Inc.) on a 96-well plate. Results were analyzed using iQ5 software (Bio-Rad Laboratories, Inc.). The universal bacterial primers 926F (5′-AAACTCAAAKGAATTGACGG-3′) and 1062R (5′-CTCACRRCACGAGCTGAC-3′) that target the 16S rRNA gene were used based on a previous study by Bacchetti De Gregoris *et al*. [34]. Each 10-μl reaction contained the following: 5 μl of ABsolute qPCR Master Mix (ABgene), 0.1 μl bovine serum albumin (10 μg μl^-1^; Thermo Scientific), 0.3 μl of each primer (10 μM, 300 nM final concentration; Integrated DNA Technologies), 3.9 μl H_2_O, and 0.4 μl template DNA. PCR conditions were 15 min at 95°C, followed by 40 cycles of 95°C for 15 s, 15 s at the annealing temperature of 57° C, and 72°C for 20 s.

The universal fungal primers FR1 (5’-AICCATTCAATCGGTAIT-3’) and FF390 (5’-CGATAACGAACGAGACCT-3’) that target the 18S rRNA gene were used based on previous studies [31,35]. Each 10-μl reaction contained the following: 5 μl of ABsolute qPCR Master Mix (ABgene), 0.1 μl bovine serum albumin (10 μg μl^-1^; Thermo Scientific), 0.1 μl of each primer (10 μM, 100 nM final concentration; Integrated DNA Technologies), 4.3 μl H_2_O, and 0.4 μl template DNA. PCR conditions were 15 min at 95°C, followed by 40 cycles of 95°C for 15 s, 30 s at the annealing temperature of 50° C, and 72°C for 1 min.

### qPCR Standards

External, fixed standards were created by amplifying and quantifying bacterial and fungal DNA using primer sets that are targeted at the full 16S/18S rRNA sequence. Template DNA extractions for bacterial and fungal standards were kindly provided from cultures of *Bacillus pumilus* (W. Hook, Grice Marine Laboratory, College of Charleston, Charleston, SC) and *Phytophthora capsici* (J. Ikerd, U.S. Vegetable Laboratory, USDA, ARS, Charleston, SC), respectively. For bacteria, the universal primers 8F (5’-AGAGTTTGATCCTGGCTCAG-3’) and 1492R (5’–GGTTACCTTGTTACGACTT-3’) were used with the following PCR conditions: 3 min at 95°C, followed by 30 cycles of 95°C for 1 min, 1 min at the annealing temperature of 45° C, and 72°C for 1.5 min, with a final elongation step of 4 min at 72°C [36,37]. For fungi, the universal primers NS1 (5’-GTAGTCATATGCTTGTCTC-3’) and NS8 (5’–TCCGCAGGTTCACCTACGGA-3’) were used with the following PCR conditions: 4 min at 95°C, followed by 30 cycles of 95°C for 1 min, 2 min at the annealing temperature of 55° C, and 72°C for 1.5 min, with a final elongation step of 10 min at 72°C [38,39]. Each 25-μl PCR reaction contained the following: 5 μl of buffer (5X colorless GoTaq Flexi Buffer, Promega), 3 μl of MgCl_2_ (25 mM; Promega), 0.25 μl bovine serum albumin (10 μg μl^-1^; Promega), 0.5 μl dNTP (10mM; Thermo Scientific), 0.5 μl of each primer (5 μM; Integrated DNA Technologies), 0.1 μl of DNA polymerase (5 U μl^-1^; GoTaq Flexi DNA polymerase, Promega), 13.15 μl H_2_O, and 2 μl template DNA. Bacterial and fungal PCR products were then run on a 0.5% agarose gel, purified with a Qiaquick Gel Extraction Kit (Qiagen), and eluted in 30 μl at the final step. Standards were quantified using the Qubit dsDNA BR Assay Kit (Invitrogen, Life Technologies) and a Qubit 2.0 Fluorometer (Qubit Systems) to determine the quantity of DNA in the final sample. The amplicon length was then used to calculate the number of copies per μl, under the assumption that the molecular weight of each bp is 650. A standard curve was generated using 10-fold dilutions of these standards across 8 orders of magnitude, therefore standardizing qPCR results by copy number.

Each plate included triplicate reactions per DNA sample and for the standard curve, as well as a no-template control to check for contamination. A melt curve (1 min at 95°C, 1 min at 55°C, +0.5°C 10 s^-1^ to 95°C) was used at the end of each qPCR run to ensure the fluorescence signal resulted from specificity to the PCR product rather than from primer dimers or other non-specific products. The fluorescein dye included in the master mix allowed for a standard normalization of error across samples. Threshold cycles (Ct) were automatically calculated by the software based on the average background noise. Samples with less than one order of magnitude of separation (3.3 Ct value) from the no-template control were also excluded given that this was outside of the detection limit of the present analysis [40,41].

### Statistical Analysis

Statistical analyses were conducted using JMP 10 (SAS Institute, www.jmp.com). Hatching success, hatchling mass, temperature, pO_2_, bacterial and fungal abundance, water content, organic matter content, and mean grain size data were all analyzed using ANOVA. When assumptions of normality or homogeneity of variance were not met, nonparametric tests were used (Wilcoxon/Kruskal-Wallis). Treatment types (removal vs. topical) were also grouped together for comparison. Given that there was no statistical difference in oxygen or temperature in no-nest controls (n = 5) across treatments, these were grouped together by treatment type (n = 15) for statistical analyses and visualization purposes. Statistically significant results were further tested with a Tukey’s HSD post-hoc test to determine significant differences between each experimental treatment. Relationships between variables (hatching success, hatchling mass, pO_2_, temperature, bacterial and fungal abundance, grain size, loss in organic matter content, and water content) were evaluated using Spearman’s rank correlation coefficients.

We used a Generalized Linear Model (GLM) to determine which factors influenced hatching success in our study. The factors that were expected to influence hatching success were treatment, treatment type, mean nest pO_2_ in the first and second half of incubation, mean nest temperature in the first and second half of incubation, the number of days above the lethal temperature limit (35°C), the loss in organic matter content in the sand over the incubation period, and bacterial and fungal abundance. We also included the combined effect (interaction terms) of both bacterial and fungal abundance with nest pO_2_ and temperature in the first and second half of incubation as well as their combined effect with the loss in organic matter content in the sand over the incubation period. We ran this full model and used a stepwise algorithm using the Akaike information criterion (AIC) to choose the best reduced model, with a lower AIC value indicating the more parsimonious model. We ran this GLM analysis using the program R (The R Foundation for Statistical Computing, 2014).

Because the embryonic development of sea turtles is limited within the first half of incubation, differences in nest oxygen content and temperature within this time period can presumably be attributed to microbial activity [11,42]. Mean nest pO_2_ and temperature were therefore analyzed for the first and second half of incubation separately. Repeated measurements of temperature and oxygen were also analyzed with repeated measures MANOVA. A χ^2^ test was also performed to test whether there were significantly more nests with hatching success below the overall mean (42%) when comparing nests that reached the lethal temperature limit (35°C) to those that did not. To compare samples across qPCR plates, an ANCOVA was used to ensure there was no significant difference between standard curves from different runs. We also used a positive control on each plate to calculate a coefficient of variation (CV) to ensure reproducibility within and between all plates. All values are expressed as means ± standard error (SE). Percentage data (hatching success) and microbial abundance data (copy number) were arcsine and log-transformed, respectively, before analysis with parametric statistics. The loss in organic matter content was calculated by subtracting the initial sampling time point (nest relocation) from the final sampling time point (nest excavation). Mean grain size was converted from phi units (ϕ) to mm for presentation. The number of 16S/18S copies ul^-1^ of template was converted to a number of 16S/18S copies g^-1^ of nest sand to allow for comparison across samples. All analyses were tested for statistical significance at α < 0.05.

## Results

The sample *arribada* occurred in October 2013 (estimated at 186,076 egg-laying females) and another *arribada* (November 2013; estimated at 110,263 egg-laying females) occurred before excavations were carried out at the end of December (R. Valverde, pers. obs.). Nest density for the area of the beach adjacent to the hatchery at the time of excavation was approximately 1.6 nests m^-2^ and hatching success for *in situ* nests (not from this study) laid on this same area of the beach during the October *arribada* was approximately 27% (R. Valverde, pers. obs.). Hatching success for study nests located in sand with no treatment was similar (32%) to that of natural *in situ* nests (unpaired *t*-test, *t* = 1.132, df = 35, p = 0.276).

### Hatching Success

**Fig 1:**
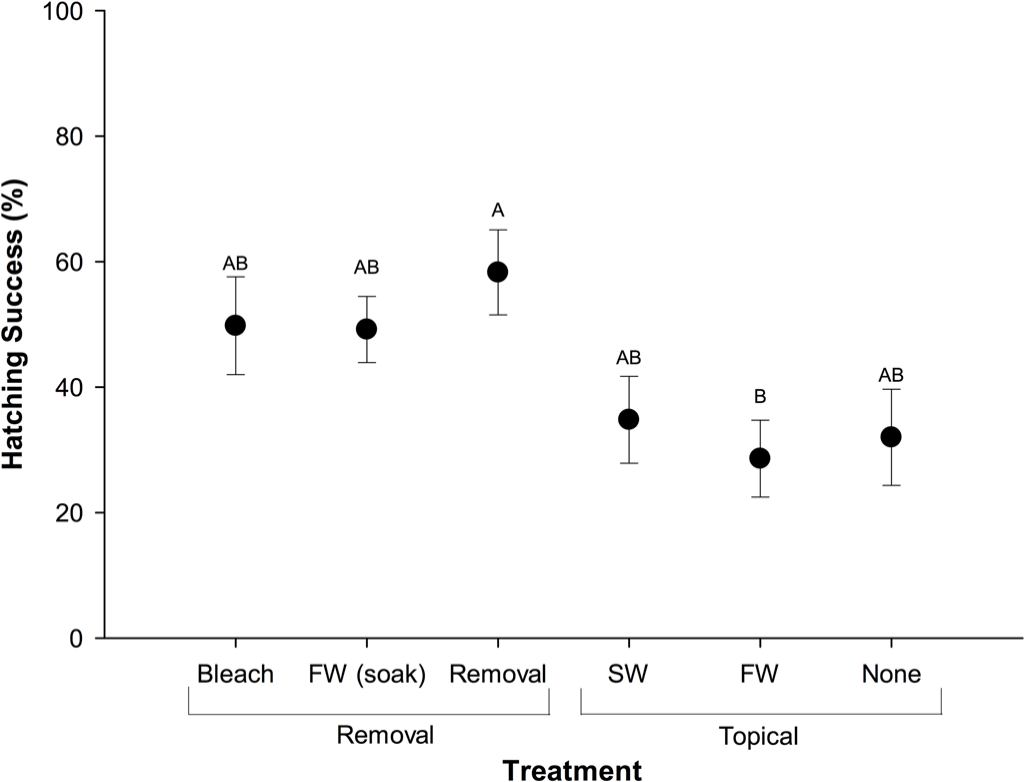
Mean (± SE) hatching success [hatchlings clutch^-1^ (%)] for nests relocated into experimental plots. n = 10, FW = freshwater, SW = seawater.

**Fig 2:**
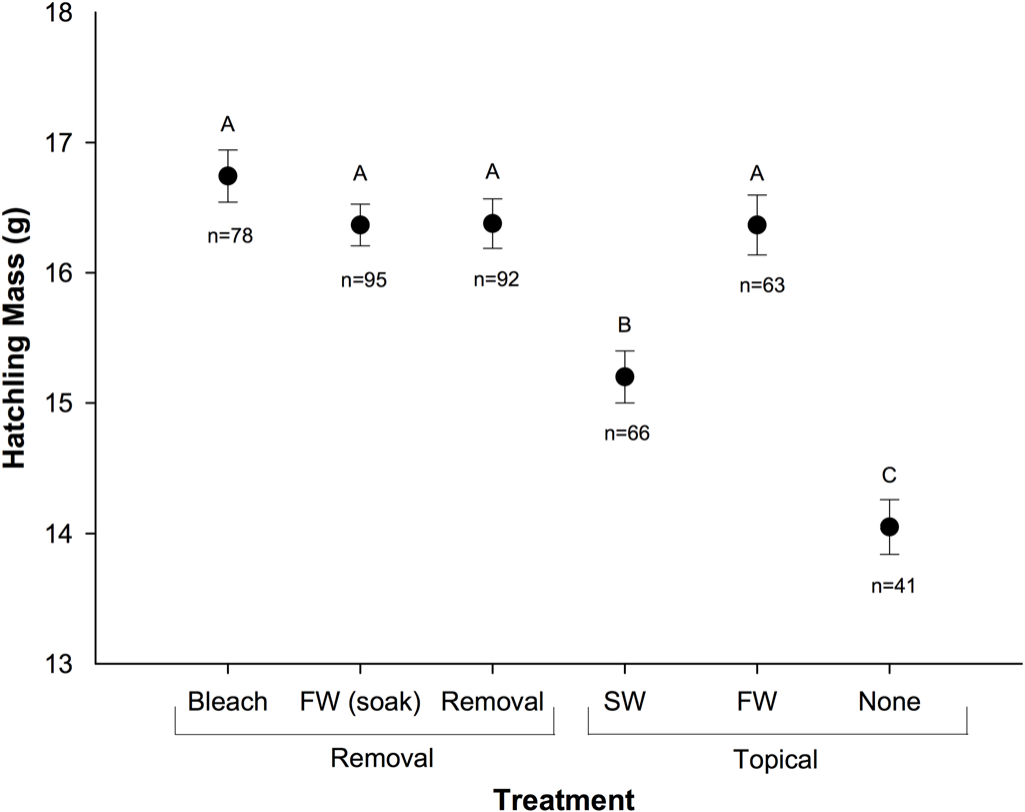
Mean (± SE) mass of hatchlings from nests relocated into experimental plots. n = number of hatchlings weighed, FW = freshwater, SW = seawater.

There was a significant effect of treatment on hatching success (p = 0.015; Fig. 1, S1 Table) and removal treatments had significantly higher hatching success (52%) than topical treatments (32%, p = 0.001). Additionally, there was a significant effect of treatment on hatchling mass (p = 0.003; Fig. 2, S2 Table). Hatchlings from nests located in sand topically treated with seawater (15.2 ± 0.4 g) and untreated sand (14.1 ± 0.2 g) weighed significantly less than all other treatments. Hatching success was positively correlated with hatchling mass (r = 0.554, p < 0.001). The mean duration of the incubation period across all treatments was 51.5 ± 0.2 d (range 49–55 d).

Results from the Generalized Linear Model statistical analysis provided a model of best fit (AIC = -22.027) with the significant main effects influencing hatching success being no treatment to the nest sand (p = 0.030), pO_2_ in the first half of incubation (p = 0.013), temperature in both the first and second half of incubation (p = 0.030 and p < 0.001, respectively), the number of days above the lethal temperature limit (p = 0.030), fungal abundance (p = 0.025), and the combined effect of bacterial abundance with temperature in both the first and second half of incubation (p = 0.030 and p < 0.001, respectively) as well as the combined effect of fungal abundance with pO_2_ in the first half of incubation (p = 0.022). The model of best fit also included all other treatments, the loss in organic matter content over the incubation period, bacterial abundance, and the combined effect of fungal abundance and the loss in organic matter content in the sand over the incubation period, although these did not have significant effects on hatching success on their own (p > 0.100 for all such effects).

### Nest pO_2_

**Fig 3:**
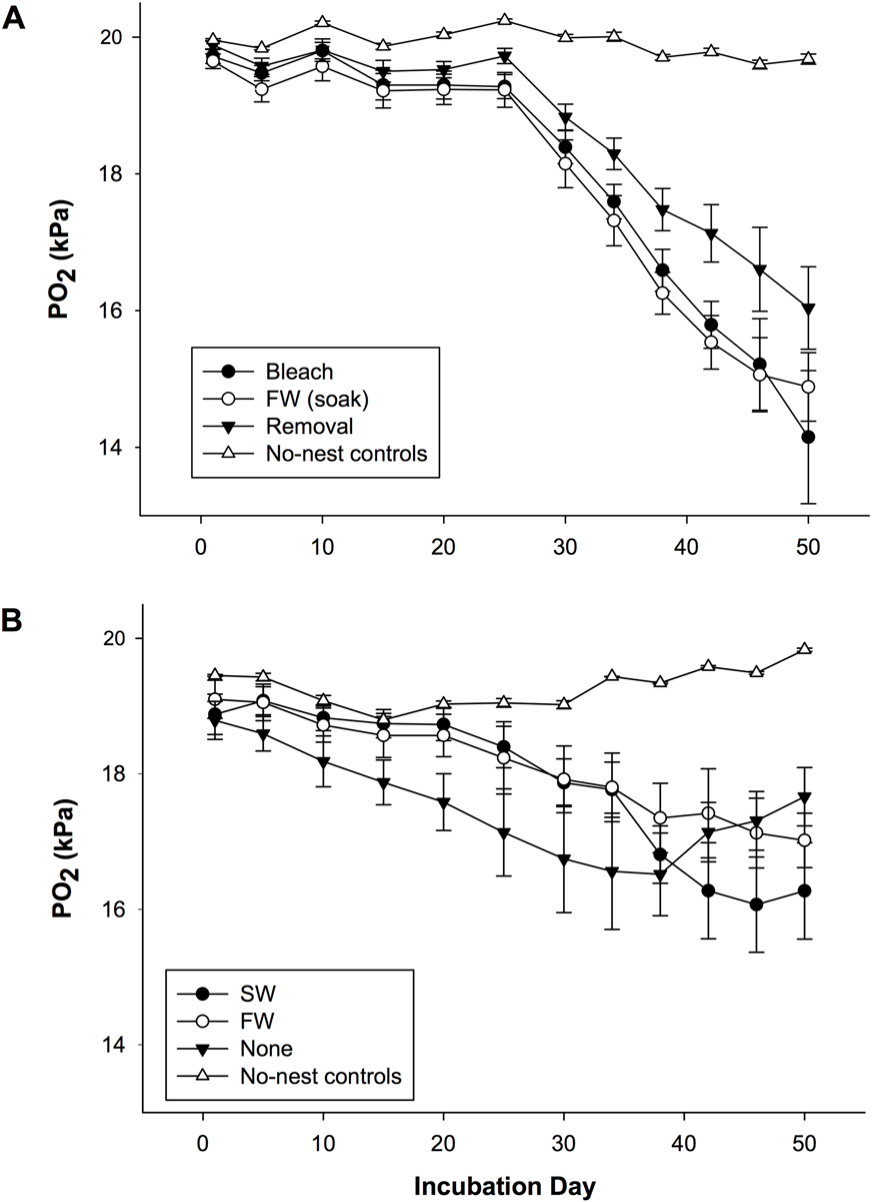
Mean (± SE) partial pressure of oxygen (kPa) in nests relocated into (A) removal treatments and (B) topical treatments. n = 10 nests or 15 no-nest controls, FW = freshwater, SW = seawater.

There was a significant effect of treatment on mean nest pO_2_ over the first half of incubation (p < 0.001), but not over the second half of incubation (Fig. 3, S3 Table). Nests located in sand with no treatment had significantly lower pO_2_ in the first half of incubation than all removal treatments (bleach, p = 0.001; freshwater (soak), p = 0.003; removal, p < 0.001).

The pO_2_ in nests from topical treatments (18.50 ± 0.18 kPa) was significantly lower in comparison to nests from removal treatments (19.50 ± 0.09 kPa; p < 0.001) in the first half of incubation. There was not a significant effect of treatment on the pO_2_ in nests over the entire incubation period. However, there was an effect of incubation day and an interaction effect of incubation day and treatment on nest pO_2_ (Table 1, S3 Table). There was also a significant effect of treatment type (removal vs. topical) on nest pO_2_ for the entire duration of incubation (Table 2, S3 Table). Moreover, there was a positive correlation between hatching success and nest pO_2_ in the first half of incubation (Table 3, S1 Table).

**Table 1:** Summary of results from the repeated measures MANOVA investigating the effect of treatment (bleach, freshwater soak, removal, seawater, freshwater, none), incubation day (sampling date), and the combined effect of these two variables (treatment × incubation day) on nest partial pressure of oxygen (pO_2_) and temperature throughout the incubation period.

	pO_2_			Temperature		
Source	d.f.	*F*	*P*	d.f.	*F*	*P*
Treatment	5, 84	1.9346	0.097	5, 84	0.6188	0.686
Incubation Day	10, 75	22.9543	< 0.001	49, 36	429.4768	< 0.001
Treatment × Incubation Day	50, 345.42	3.1578	< 0.001	245, 184	2.6320	< 0.001

**Table 2:** Summary of results from the repeated measures MANOVA investigating the effect of treatment type (removal, topical, no-nest control), incubation day (sampling date), and the combined effect of these two variables (type × incubation day) on nest partial pressure of oxygen (pO_2_) and temperature throughout the incubation period.

	pO_2_			Temperature		
Source	d.f.	*F*	*P*	d.f.	*F*	*P*
Treatment type	2, 87	36.0547	< 0.001	2, 87	25.4783	< 0.001
Incubation Day	10, 78	35.5453	< 0.001	49, 39	417.8555	< 0.001
Type × Incubation Day	20, 156	10.0713	< 0.001	98, 78	35.1716	< 0.001

**Table 3:** Relationships between hatching success and partial pressure of oxygen (pO_2_) and temperature in the first (1^st^) and second (2^nd^) half of incubation, bacterial and fungal abundance (16S/18S rRNA gene copies g^-1^ nest sand), loss in organic matter content, and water content of the sand for nests located in different experimental treatment plots.

		Hatching Success		
Variable	Half of incubation	*r*	*n*	*P*
pO_2_	1^st^	0.622	60	**< 0.001**
	2^nd^	0.044	60	0.739
Temperature	1^st^	-0.337	60	**0.009**
	2^nd^	-0.133	60	0.312
Bacterial abundance		-0.238	60	0.067
Fungal abundance		-0.598	60	**< 0.001**
Loss in organic matter content		-0.156	60	0.239
Water content		-0.043	60	0.748

*r*, Spearman’s rank correlation coefficient; *n*, sample size. Values in bold indicate statistically significant correlations.

### Nest Temperature

**Fig 4:**
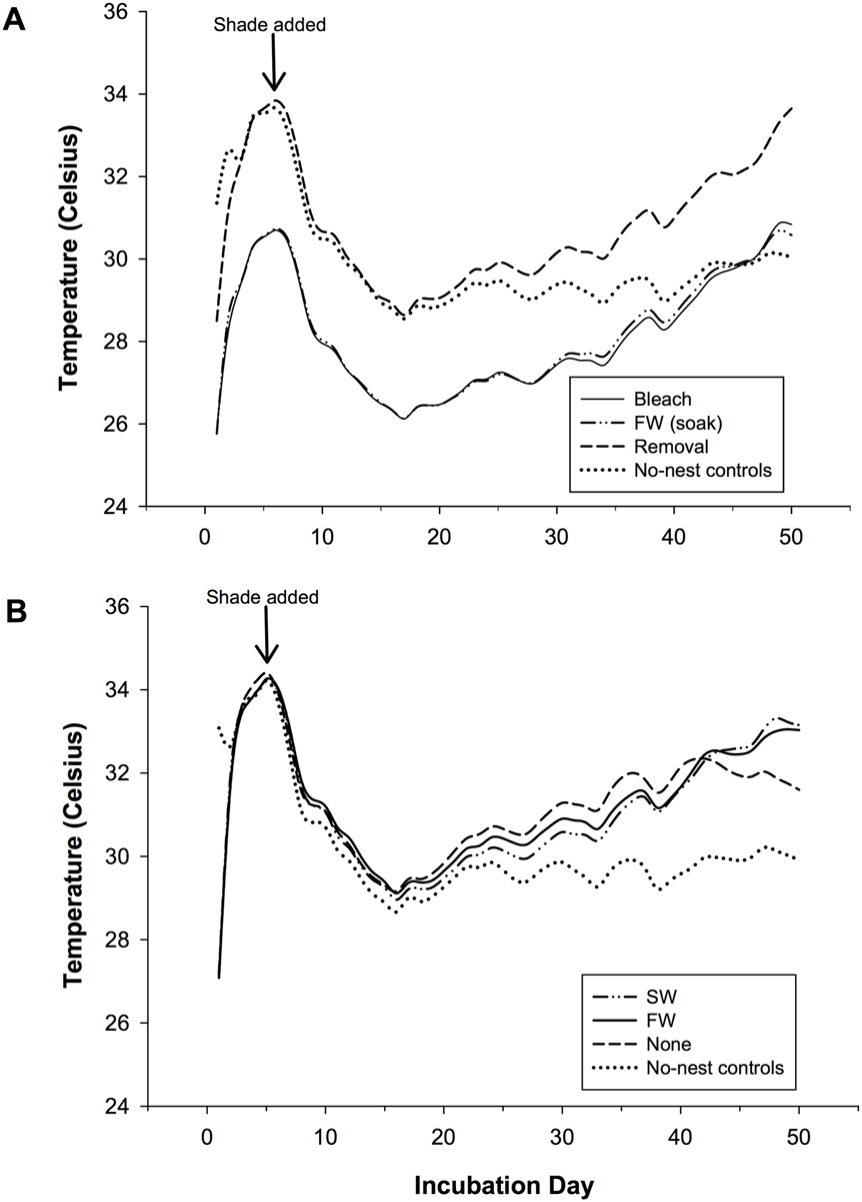
Mean nest temperature in nests located in (A) removal treatments and (B) topical treatments. n = 10 nests or 15 no-nest controls, FW = freshwater, SW = seawater.

There was not a significant effect of treatment on nest temperature in the first and second half of incubation (Fig. 4, S4 Table). However, removal treatments had a significantly lower temperature (30.51 ± 0.05°C) than topical treatments (30.78 ± 0.10°C) in the first half of incubation (p = 0.015). There was no effect of treatment on nest temperatures over the entire incubation period, though there was an effect of incubation day as well as an interaction between these two factors (Table 1, S4 Table). On the other hand, there was a significant effect of treatment type (removal vs. topical) and incubation day on nest temperature as well as an interaction between these two factors (Table 2, S4 Table). Mean daily nest temperatures in most nests did not exceed the lethal limit (35°C). However, nest temperatures in 17 nests just barely exceeded this limit (0.89 ± 0.12°C) at the end of the incubation period (day 40.88 ± 1.90 of incubation) and few for extended periods of time (1.06 ± 0.30 d, range 0–11 d). Nests that reached the lethal temperature limit had lower hatching success than the overall mean (p = 0.037). However, these nests were evenly distributed throughout the experimental set-up and treatments and therefore were not associated with any particular treatment. There was a negative correlation between hatching success and nest temperature in the first half of incubation (Table 3, S1 Table). Additionally, there was a negative correlation between the duration of the incubation period and temperature in both the first and second half of incubation (r = -0.604, p < 0.001; r = -0.733, p < 0.001).

### Composition of Nest Sand

**Fig 5:**
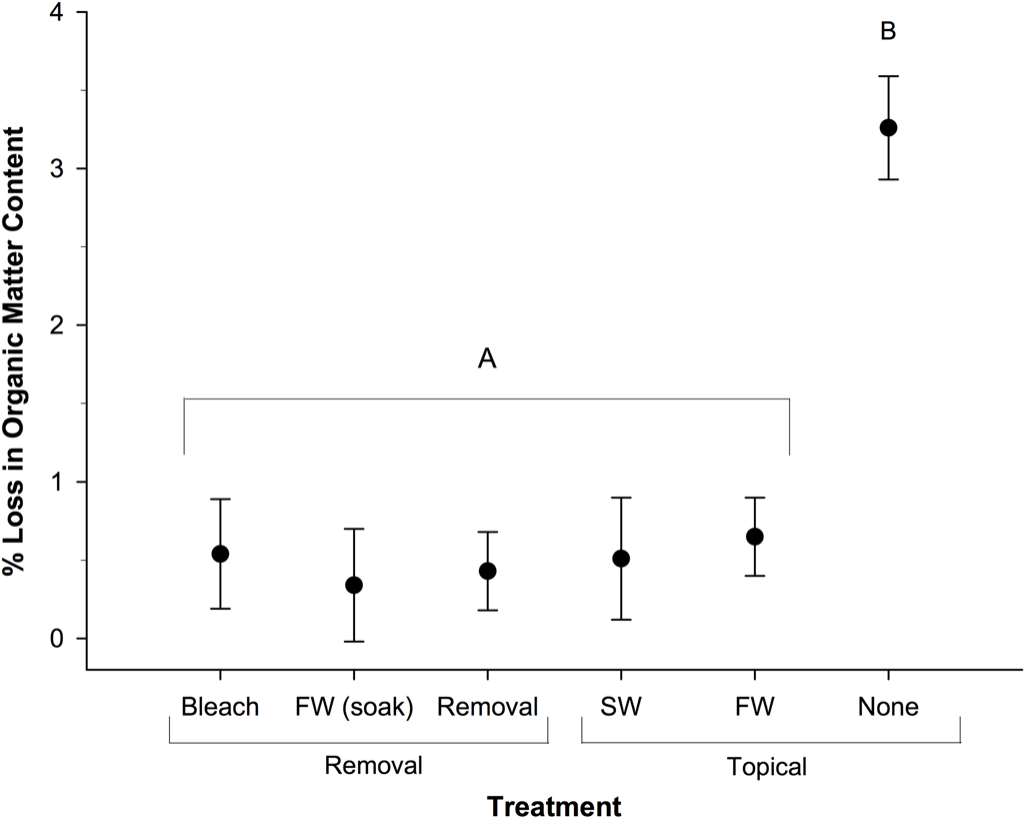
Mean (± SE) percent loss in organic matter content in sand from nests located in the different experimental treatments. n = 10, FW = freshwater, SW = seawater.

There was a significant effect of treatment on the loss of organic matter content of nest sand over the incubation period (p < 0.001, Fig. 5, S1 Table). Sand from nests located where there was no treatment had the greatest loss in organic matter content (3.26 ± 0.33%) in comparison to sand from nests located in all other treatments (0.49 ± 0.14%). On the other hand, there was no significant difference in water content (4.58 ± 0.10%) across treatments for nest sand at the final sampling time point. Nest sand did not differ in granulometric properties across treatments. The nest sand from all study nests had a mean grain size of 0.298 ± 0.004 mm, was poorly sorted (σ_ϕ_ = 1.072 ± 0.013), and coarse skewed (*Sk*_ϕ_ = -0.515 ± 0.052). There was no significant correlation between the loss in organic matter content or the water content of nest sand and hatching success (Table 3, S1 Table).

### Microbial Abundance

There was no significant difference in the slope of the standard curve across assays for bacteria (*E =* 90.78 ± 0.41%, R^2^ = 0.99 ± 0.01, slope = -3.56 ± 0.01, intercept = 38.81 ± 0.45, CV = 3.87%) or fungi (*E =* 90.88 ± 1.20%, R^2^ = 1.00 ± 0.00, slope = -3.56 ± 0.03, intercept = 46.01 ± 0.22, CV = 2.48%), indicating that copy numbers were comparable across assays (p = 0.992 and p = 0.905, respectively). Microscopy counts (range 3.11 × 10^6^–2.56 × 10^9^ cells g^-1^ of nest sand) were significantly correlated with qPCR results for 16S rRNA gene copy number g^-1^ of nest sand (r = 0.485, p < 0.001). The negative controls for each set of DNA extractions all fell within 3.3 Ct values of the no-template control for qPCR, indicating there was no significant signal originating from the extraction process alone. All other samples fell within the detection limit of the present analyses at a 1:100 and 1:10 dilution for bacteria and fungi, respectively.

**Fig 6:**
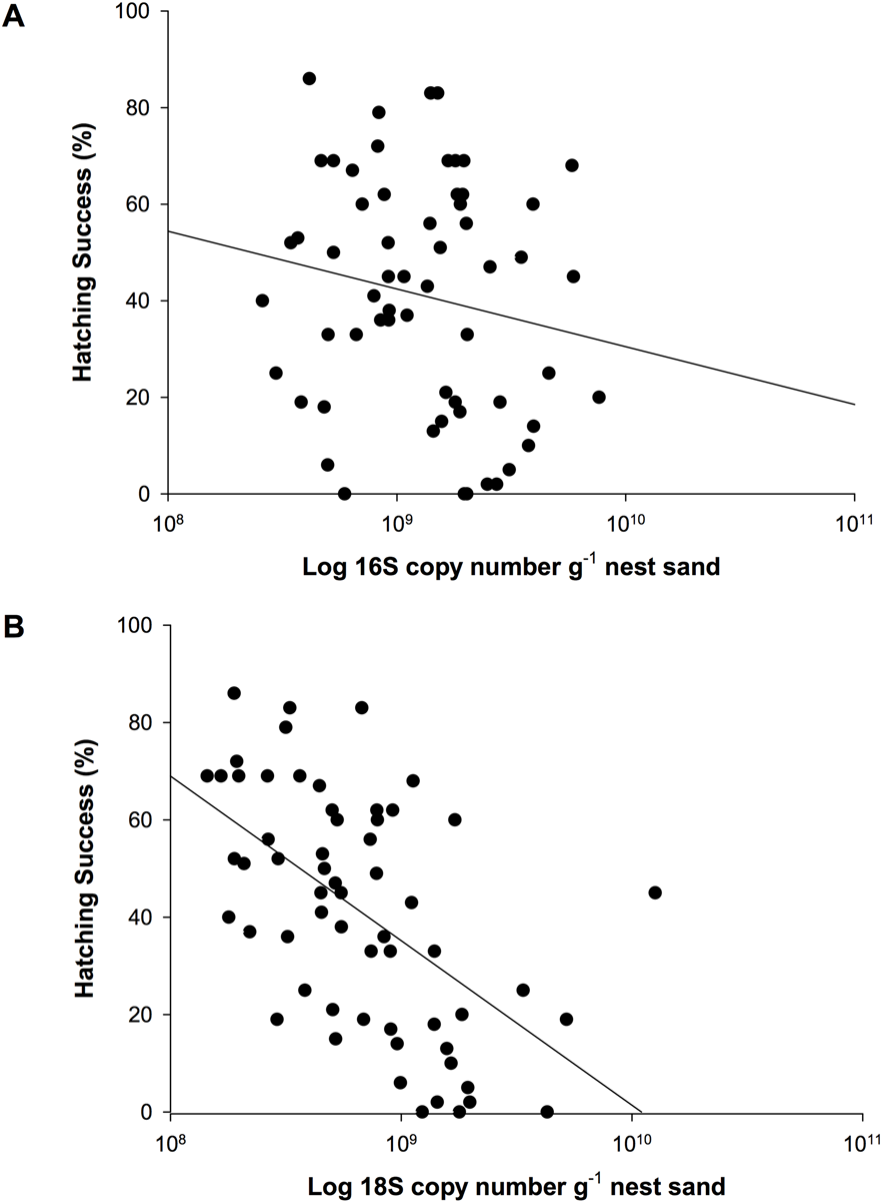
The relationship between hatching success and microbial abundance (log scale). **(A) Bacterial 16S and (B) Fungal 18S rRNA gene copy number g** ^**-1**^ **nest sand**. n = 60.

There was a significant effect of treatment on the fungal abundance in nest sand, however there was no effect of treatment on bacterial abundance (p = 0.002 and p = 0.263, respectively). Specifically, nest sand with no treatment had copies of the fungal 18S rRNA gene totaling an order of magnitude greater than sand that underwent the removal treatment (p = 0.002). Nest sand from removal treatments also had fungal 18S copy numbers an order of magnitude less than sand from topical treatments (p < 0.001). On the other hand, there was no apparent effect of treatment type on bacterial abundance (p = 0.339). The correlation between hatching success and bacterial abundance was marginal (p = 0.067), yet there was a strong negative correlation between hatching success and fungal abundance (Table 3, Fig. 6, S1 Table). Additionally, there was a negative correlation between microbial abundance (both bacteria and fungi) and nest pO_2_ as well as a positive correlation with nest temperatures in both the first and second half of incubation (Table 4, S1 Table). There was no significant correlation between either bacterial or fungal abundance and the loss in organic matter content in nest sand (r = -0.176, p = 0.183; r = 0.153, p = 0.277; respectively).

**Table 4:** Relationships between the abundance of bacteria (16S rRNA gene copies g^-1^ nest sand) and fungi (18S rRNA gene copies g^-1^ nest sand) and the partial pressure of oxygen (pO_2_) and temperature in the first (1^st^) and second (2^nd^) half of incubation for nests located in the different experimental treatment plots.

		pO_2_				Temperature			
Half of Incubation		1^st^		2^nd^		1^st^		2^nd^	
	*n*	*r*	*P*	*r*	*P*	*r*	*P*	*r*	*P*
Bacteria	60	-0.37	**0.003**	-0.67	**< 0.001**	0.297	**0.021**	0.459	**< 0.001**
Fungi	60	-0.66	**< 0.001**	-0.25	0.056	0.35	**0.006**	0.21	0.107

r, Spearman’s rank correlation coefficient; n, sample size. Values in bold indicate statistically significant correlations.

## Discussion

The negative relationship between both bacterial abundance and fungal abundance and hatching success in study nests supports the hypothesis that the microbial abundance in nest sand at Ostional is high enough to adversely affect embryonic development. Specifically, the results support the hypothesis that the mechanism behind this relationship is a result of high rates of microbial decomposition altering the nest environment beyond the optimal range for embryonic development [6,7,11]. In particular, our GLM analysis suggests that the combined effect of high nest temperatures and low nest pO_2_ early in incubation resulting from the microbial decomposition of organic matter in the nest are influential in determining hatching success at Ostional. Additionally, based on both our correlation and GLM analyses, fungi seem to play a larger role than bacteria in altering the nest environment and influencing hatching success. Furthermore, our results support the hypothesis that the treatment of sand in ways that are likely to alter microbial abundance and diversity has a positive effect on hatching success.

### Hatching Success

The overall hatching success in this study (42%) was low in comparison to that observed for olive ridley sea turtles at a solitary nesting beach (74–81%; [4,5]), yet comparable to previous studies at this and other mass nesting beaches [2,3,6]. However, hatching success was higher in all of our treatment plots (42%) in comparison to the area of the beach adjacent to the hatchery (nests not from this study, 27%; R. Valverde, pers. obs.). Although it is possible that the addition of shade over experimental nests could have protected embryos from lethal incubation temperatures, thus yielding higher overall hatching success in comparison to unprotected *in situ* nests located in the adjacent beach, the difference in hatching success is most likely attributable to the treatment of sand. In fact, hatching success for study nests located in sand with no treatment (i.e., shaded but no sediment manipulation) was similar (32%) to that of natural nests.

Among experimental nests, the removal and replacement of nest sand was a distinguishing factor that had a positive impact on hatching success. In particular, nests relocated into the removal treatment (sand that was removed and replaced only) had the highest hatching success in comparison to all other experimental treatments. In general, nests relocated into treatments that involved the removal and replacement of sand (removal treatments) had higher hatching success than topical treatments in which the sand was not displaced. Though the exact mechanisms behind this higher hatching success remain to be identified, our data suggest that this is likely due to the disturbance of sand decreasing microbial abundance and respiration rates, ultimately resulting in higher pO_2_ in the nest environment.

While all study nests were incubating simultaneously and under the same environmental conditions, there are several factors that may have impacted the efficacy of the treatments or caused incongruences in nest conditions, potentially affecting overall hatching success in this study. Although we successfully relocated all actively incubating nests out of the study area, the heterogeneous composition of the sand at Ostional made it difficult to provide homogenous beach sand for the incubation of study nests. Due to the continuous and copious input of eggs from sea turtles at Ostional, the sand consists of patches of old, decaying eggshells at various depths, resulting in spatially heterogeneous nest conditions for sea turtles. For the purposes of this study, we chose to limit the manipulation of sand to preserve the natural conditions of the beach. Nonetheless, the Latin square design of our experimental treatment plots was intended to provide a spatial interspersion of nests such that these heterogeneous nest conditions would be evenly distributed across treatments.

### Nest pO_2_

The pO_2_ for most nests was consistent with that previously observed in other studies on olive ridley sea turtles [6,11]. Those studies also observed a significant effect of incubation day on the oxygen content of nests, with oxygen decreasing steeply in the second half of incubation in conjunction with the increasing metabolic rates associated with this stage of embryological development [11,43]. The significant combined effect of treatment (and treatment type) and incubation day on the pO_2_ of nests suggests that the pO_2_ of nests fluctuated differently across treatments. This is likely a result of the difference in the number of metabolizing embryos (and the microbial activity) across treatments as previous studies have found a correlation between nest pO_2_ and the number of metabolizing embryos per clutch [11,27].

The pO_2_ of nests located in plots with no treatment (no manipulation to the sand at all) was not comparable to that of previous studies at other solitary and mass nesting beaches. The drop in pO_2_ observed within the first half of incubation is atypical given that the metabolic activity of the sea turtle embryos is relatively low during the first half of incubation [44]. The pO_2_ in sea turtle nests is typically stable until the second half of incubation, when it decreases steeply [11]. However, we have previously observed similar trends (i.e., low pO_2_ in both the first and second half of incubation) in the oxygen levels of *in situ* nests at Ostional [45]. This difference in pO_2_ is therefore likely attributable to the difference in microbial respiration in the sand. In fact, the significant combined effect of pO_2_ and fungal abundance on hatching success in the GLM analysis suggests that fungi were likely responsible for these differences in nest pO_2_ early in incubation. Collectively, these results showcase the unique physiological conditions present for nests incubating at Ostional, where high organic matter content in the nest substrate drives relatively high rates of microbial oxygen consumption.

The positive relationship between hatching success and pO_2_ in the first half of incubation along with the significant influence of pO_2_ in the first half of incubation in determining hatching success in the GLM analysis suggests that the depletion of oxygen early in incubation likely interfered with sea turtle embryo development in this study. Previous studies found that embryological development slowed and mortality increased for sea turtle embryos exposed to environments with pO_2_ below those found naturally [10,43]. Ackerman [10] proposed that this serves as an adaptive response with decreases in oxygen consumption allowing for the survival of at least some eggs. However, in extreme cases where oxygen falls below a critical threshold, this may lead to total nest failure. A study on several species of reptiles found that sea turtle embryos have a relatively low tolerance to hypoxia, especially in the first half of incubation [46]. Though the critical oxygen tension of olive ridley sea turtle eggs remains to be investigated, their size and mass are comparable to that of loggerhead sea turtle (*Caretta caretta*) eggs, which have a critical oxygen tension as high as 16.5 kPa on day 22 of incubation [46]. It is therefore likely that eggs located in sand with no treatment were exposed to pO_2_ below this threshold given that mean nest pO_2_ fell below 16.5 kPa on day 30 of incubation. On the other hand, the potential adverse effects of hypercapnia on embryological development cannot be ruled out, although the present study did not measure CO_2_ in nests nor has this been studied in olive ridley sea turtles specifically.

### Nest Temperature

The mean and ranges of nest temperatures observed in this study are comparable with previous studies on olive ridley sea turtles in this region [6,11,26]. Previous studies have also observed a significant effect of incubation day on the temperature of nests, with temperatures increasing during the second half of incubation in accordance with increases in embryonic metabolism [6,11]. Nest temperatures were comparable across the different treatments for the entire duration of the incubation period, with the exception of nests located in the two removal treatments, where lower nest temperatures likely resulted from the treatment process. This difference in temperature likely resulted in slower embryonic development [47]. In fact, the incubation period was negatively correlated with temperature in both the first and second half of incubation for all study nests. While most nest temperatures fell within the wide range of tolerance for sea turtle embryological development (25–35°C; [8]), the GLM analysis suggests that both nest temperatures and the combined effect of bacterial abundance and nest temperature still had a significant influence on hatching success. In fact, small increases in temperature within the nest may have a greater impact on microbial respiration and decomposition given the Q_10_ response pattern that characterizes these processes [21].

The consistency in nest temperatures in the first half of incubation suggests that the small differences in temperature across treatments in the second half of incubation were likely a result of differences in the combination of heat released from embryonic metabolism and microbial decomposition. While the field lethal limit for olive ridley sea turtles at this beach is 35°C, lethality is believed to be associated with the duration of time at high temperatures rather than a threshold [26]. Lower hatching success (31%) was observed in the nests in the present study that reached this temperature, although this was not necessarily associated with a treatment (at least one nest from every treatment reached 35°C). The GLM analysis also found that the number of days that a nest experienced temperatures above 35°C was a significant factor in determining hatching success.

### Composition of Nest Sand

The mean grain size of sand from nests in this study was within the range of those previously observed at a variety of sea turtle nesting beaches [9]. Given that there was no difference in the mean grain size of nest sand across the different experimental treatments, grain size was not likely a determining factor impacting hatching success.

The organic matter content of nest sand throughout this study was higher than previously studied sea turtle nesting sites [9]. However, the high organic matter content is not surprising as this is likely a result of eggs broken by nesting females, as previously suggested [48–50]. In fact, a previous study on the nutrient composition of the sand at Ostional found ammonia, nitrate, and phosphate levels much higher than at neighboring beaches [49]. The relatively high organic matter content that we observed in comparison to other nesting beaches may explain the decreases in pO_2_ early in incubation in comparison to previous studies. In particular, the significant loss in organic matter content over the incubation period observed in nests located in untreated sand suggests higher rates of microbial decomposition. While there was not a significant correlation between microbial abundance and the loss of organic matter, this is likely a result of the small mass of sand analyzed in conjunction with the high variation in the organic matter content of the sand, which precluded a robust test. The additional source of organic matter from sea turtle eggs is likely an important factor in driving microbial activity in the sand at Ostional, although the potential effect of organic matter build-up on the diffusion of oxygen through the nest substrate cannot be ruled out.

### Microbial Abundance

The bacterial 16S and fungal 18S rRNA gene copy numbers from this study ranged from 10^8^ to 10^10^ g^-1^ of sea turtle nest sand. To our knowledge, no previous study has provided quantitative data on the abundance of bacteria or fungi in sea turtle nest sand. A previous study based on culture-dependent techniques found that the sand at an *arribada* beach contained six orders of magnitude more bacterial colonies g^-1^ than a neighboring beach, although no specific abundance data were provided [14,18]. Previous studies that have quantified 16S or 18S rRNA gene copies in similar sediments have found between 10^6^–10^9^ g^-1^ [31,51].

The negative correlations between microbial abundances and nest pO_2_ suggest that microbial decomposition was responsible for the decreases in the partial pressure of oxygen in nests. However, fungi seem to play a larger role than bacteria in altering the nest environment and influencing hatching success. In fact, our GLM analysis suggests that the decomposition of organic matter and consumption of oxygen by fungi in particular was a driving factor in decreasing hatching success. While bacteria are fairly limited within sediments that have a patchy distribution of organic matter, fungal hyphae have the capacity to reach organic matter trapped within sediment pores and may therefore be more active players in the decomposition of organic matter in heterogeneous sediments [23]. Because the ratio of rRNA gene copies per unit of biomass is much lower for fungi in comparison to bacteria, the relative biomass of fungi in nest sand was likely greater than that of bacteria given the copy numbers observed in this study [52]. Fungal biomass is typically greater than bacterial biomass in a variety of systems, including soils [53,54]. Additionally, our analysis may show closer correlations with fungal abundance given that bacterial 16S copy numbers may include autotrophic organisms while our fungal 18S copy numbers exclusively represent heterotrophic organisms.

The turnover of sand stands out as a distinguishing step in the treatment process that improved hatching success in this study. This becomes apparent when comparing the removal and topical treatments, with the removal and replacement of sand as a differentiating factor between the two treatment types. The distinguishing difference in nest conditions between these treatment types was an increase in the pO_2_, particularly in the first half of the incubation period. This difference in pO_2_ could be due to the aeration of the sand from turnover and/or differences in the microbial composition of the sand and the respiration rates of microbes as a result of this step in the treatment process. Studies have found that turnover alters the microbial community composition of sediments as well as microbial respiration and decomposition rates [21–23]. Physical disturbance can decrease bacterial abundance and disfavor fungi by disrupting the fungal hyphal network [55]. In particular, sand from nests located in plots where there was no treatment (no disturbance) at all had the largest loss in organic matter over the incubation period, which is indicative of higher rates of microbial decomposition. On the other hand, the turnover of sand could release potentially toxic gases (e.g., ammonia) that have built up over time from microbial decomposition into the atmosphere and/or allow for their oxidation into inert forms. In fact, an agricultural study on tillage suggested that physical processes, rather than microbial activity, were responsible for changes in the flux of carbon dioxide from soils [21]. Ammonia levels up to 460 times greater than neighboring beaches have been observed in the sand at Ostional and previous studies on oviparous reptiles have found ammonium nitrate and ammonia fertilizer to be toxic to embryos [49,56,57]. Further studies are needed to identify the presence of potentially toxic gases in the sand at Ostional and to determine whether these gases have any effect on developing sea turtle embryos.

The results from our experimental treatments indicate that the treatment of sand could be used to improve hatching success in small-scale management practices. Many existing sea turtle conservation management practices involve the relocation of nests into hatcheries to protect them from poaching, predation, or erosion. In these cases, the treatment of sand (e.g., sifting) or relocation of the hatchery on a regular basis has become commonplace to avoid organic matter build-up as well as microbial and larval infestations. Using natural mechanisms as a model for conservation, we can simulate conditions that optimize the nest environment most conducive to successful embryological development. For example, sand turnover could serve as a feasible and effective treatment to increase hatching success, although the exact mechanism behind the observed increase in hatching success remains to be determined. The efficacy of the removal treatment, and all removal treatments for that matter, suggests that turnover of sand itself significantly improves the quality of nest sand for sea turtle embryonic development at Ostional. Further field-testing of antimicrobial treatments should be carried out to confirm the applicability of this methodology to conservation through hatchery management for beaches affected by microbial infestations.

The microbial interference of embryological development could certainly be an important selective force for the reproductive success of mass nesting females and even challenges our understanding of the evolutionary advantage of this behavior [6,11]. However, erosion events that replace and/or “clean” the sand may provide for the high hatching success of a single mass nesting event to outweigh the fitness disadvantage of high mortality in subsequent events over an evolutionary time scale [11]. Therefore, the high rates of sand turnover characteristic of mass nesting beaches is likely critical in maintaining this behavior over the long term. On the other hand, *arribada* beaches could be ephemeral, with shifts in nesting populations reflecting potentially suboptimal nesting conditions due to increasing nest densities and organic matter build-up over time [1,2,7].

The results of this study emphasize the importance of increasing our understanding of sea turtle-microbe interactions to identify and address threats imposed by anthropogenic impacts such as organic loading and climate change. Wastewater management could be critical in limiting the anthropogenic impacts of organic loading, which could influence the organic matter content of nest sand at nesting beaches near riverine outputs. Additionally, the quality of sand used in the renourishment of sea turtle nesting beaches could be fundamental to protecting the reproductive success of local populations. Because decomposition rates increase with increasing temperatures, rising global temperatures could exacerbate the detrimental effects of high decomposition rates on hatching success. Given the presently low hatching success and decrease in *arribada* nesting population abundance at Ostional [3], factors that are negatively affecting hatching success are especially important for consideration in conservation management at this beach. At beaches around the world, it is important to consider the potential effects of anthropogenic organic matter inputs and microbial decomposition on the reproductive success of sea turtles to effectively address and eliminate such threats to the conservation of the species.

## Supporting Information

S1 Table Effect of nest sand treatment and treatment type on hatching success, pO_2_, temperature, incubation period, water content, organic matter content, 16S/18S copy number g^-1^ nest sand, and microscopy count for bacteria. (XLSX)

S2 Table Effect of nest sand treatment and treatment type on hatchling mass. (XLSX)

S3 Table Effect of nest sand treatment and treatment type on nest pO_2_ throughout the incubation period. (XLSX)

S4 Table Effect of nest sand treatment and treatment type on nest temperature throughout the incubation period. (XLSX)
